# Avian influenza spillover into poultry: environmental influences and biosecurity protections^☆^

**DOI:** 10.1016/j.onehlt.2025.101172

**Published:** 2025-08-19

**Authors:** Matthew Gonnerman, Jennifer M. Mullinax, Andrew Fox, Kelly A. Patyk, Victoria L. Fields, Mary-Jane McCool, Mia K. Torchetti, Kristina Lantz, Jeffery D. Sullivan, Diann J. Prosser

**Affiliations:** a Department of Environmental Science and Technology, University of Maryland, College Park, MD 20742, USA; b Post-doctoral affiliate with the U.S. Geological Survey, Eastern Ecological Science Center, Laurel, MD 20708, USA; c United States Department of Agriculture, Animal Plant and Health Inspection Service, Veterinary Services, Strategy and Policy, Center for Epidemiology and Animal Health, Fort Collins, CO 80526, USA; d National Veterinary Services Laboratories, Animal and Plant Health Inspection Service, USDA, Ames, IA, 50010, USA; e U.S. Geological Survey, Eastern Ecological Science Center, Laurel, MD 20708, USA

**Keywords:** Avian influenza, Surveillance, Biosecurity, Waterfowl, Poultry, Risk modeling

## Abstract

With the continued spread of highly pathogenic avian influenza (HPAI), understanding the complex dynamics of virus transfer at the wild – agriculture interface is paramount. Spillover events (i.e., virus transfer from wild birds into poultry) are related to proximity to infected wild bird populations and environmental conditions. By accounting for such dynamics, we can take a combined approach to assess the impacts of biosecurity measures implemented at poultry farms while simultaneously accounting for their local risk levels. We implemented a Bayesian joint-likelihood logistic regression for the Continental U.S. comparing models of spatiotemporal risk according to land use, weather, and predicted waterfowl distributions followed by integrating a farm-level case-control questionnaire dataset focused on identifying trends in HPAI spillover risk associated with a farm's biosecurity practices. We found that estimates of waterfowl abundance, along with mean precipitation and temperature during winter, were most correlated with spatiotemporal HPAI risk. Additionally, we identified multiple biosecurity practices associated with reduced risk to HPAI, where the strongest relationships were related to litter decontamination treatments, vehicle wash stations, and avoiding shared dead-bird disposal sites with other farms. This model broadly guides surveillance of HPAI in wild and domestic populations, identifying when and where we are most likely to see increased instances of the virus while also providing insights into how poultry farms can better protect themselves from risk.

## Highlights

• Integrated case-control and spatial data to assess biosecurity efficacy in context of local risk.
• Human-mediated transmission pathways contribute to avian influenza spillover into poultry.
• On-site vehicle decontamination and providing restroom facilities reduced spillover risk.
• Litter decontamination treatments and avoiding shared dead-bird disposal sites reduced risk.

## Graphical abstract

**Figure:**
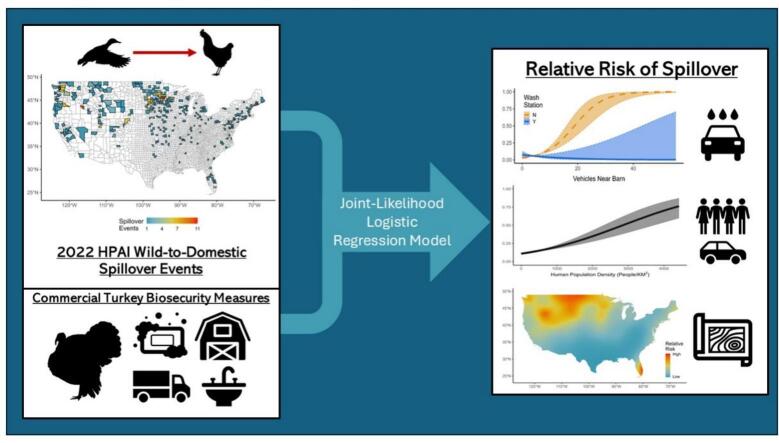

## 1 Introduction

Highly pathogenic avian influenza (HPAI) has been a conservation and human health concern for decades [1,2,3]. HPAI risk management has primarily focused on avian species, attempting to limit transmission in wild bird populations, spillover into domestic poultry (and recently dairy) operations, and spread between poultry facilities [4,5,6]. Outside of the limited outbreak in 2014–2015 in the U.S. [7,8], efforts to describe this disease system have been limited to information from outbreaks in Europe and Asia [9,10,11]. While some dynamics may repeat, the broader scale pattern of outbreaks across a continent are driven by spatial and temporal processes, such as the movements of available wild bird host species and viral subtypes in the context of each continent's unique landscape. Thus, a predictive risk model for the United States would ideally be produced using information from the U.S. system, but prior to 2021 sufficient data was not available. Following multiple incursions from Europe and Asia beginning in 2021 [12,13], North America experienced an unprecedented number of HPAI infections in wild bird populations [14], which subsequently resulted in over 500 unique instances of HPAI spilling over into poultry farms. These spillover events necessitated the culling of millions of infected poultry [15], causing considerable financial loss and disruption to supply chains [16]. Data collected from this series of outbreaks provides an opportunity to better describe the dynamics of HPAI spillover risk at the farm-level in the United States.

At broad scales, HPAI transmission is facilitated by the movements of migratory birds [17,18]. While many species are potential hosts, waterfowl are suspected to be a primary driver of transmission [19,20]. Dabbling ducks, as communal feeders which occupy freshwater aquatic ecosystems, are ideal hosts for transmitting HPAI. In challenge studies, many of these dabbling duck species exhibit high viral shedding rates via the cloaca [21], which can result in the accumulation of large viral loads and perpetuate disease spread [22]. This relationship is further supported by a greater proportion of infections in wild surveillance observations occurring in areas of high overlap between waterfowl and domestic poultry [23]. Poultry operations' exposure to HPAI is influenced by direct contact with infected animals and indirect environmental spread in areas with increased viral loads, so it may be possible to estimate operation or farm risk based on its associated waterfowl abundance or specific land use associations. Environmental persistence of the virus in cooler and wetter weather conditions [24] could further increase infection risk if conditions coincide with areas of increased waterfowl abundance, increasing and perpetuating viral loads in surface water. Given their influence on local risk levels, it may be necessary to incorporate such local and regional risk factors in assessments of farm-level risk to better account for transmission and environmental persistence of HPAI.

While spillover into poultry farms has been shown to be dependent on proximity to infected wild bird populations and environmental conditions, biosecurity measures can mitigate potential exposure for domestic flocks. This may include efforts to deter or exclude wild birds from areas where domestic flocks are housed, decontamination protocols for employees and vehicles entering the premises or barns, and ensuring that infected and dead animals are properly disposed of [25,26]. However, most poultry operations will likely not have the resources to implement all biosecurity options available, requiring some cost-benefit considerations [27]. Given these practices will vary in necessary resources or in the frequency they must be undertaken, understanding their impact can better determine efficient and effective biosecurity regimes for farms to implement and maximize protection. In response to the ongoing HPAI outbreak, United States Department of Agriculture Animal and Plant Health Inspection Service (USDA-APHIS) conducted a case-control survey, requesting information about biosecurity practices to investigate potential risk factors on commercial meat turkey operations [28]. Combining this survey with the spatiotemporal characteristics of the larger poultry-wide spillover dataset provided a novel means to assess the efficacy of biosecurity within the context of a farm's local risk level.

This study describes relative risk of HPAI H5N1 spillover into domestic poultry operations using two datasets describing phylogenetically-confirmed spillover cases: spatiotemporal information describing all confirmed wild-to-domestic spillover events [13] and a case-control survey conducted at a subset of commercial turkey farms [28]. We first generated models of environmental risk, describing observed spillover events according to waterfowl habitat and regional spatiotemporal trends. After controlling for these potentially confounding local and regional drivers, we fit a Bayesian joint-likelihood model that combines information on spillover events with case-control questionnaire data at a subset of locations. By combining the larger spatiotemporal dataset with data collected in the case-control study, we assessed correlation of spillover risk with implementation of various biosecurity measures, after accounting for the patterns associated with local environmental characteristics. From this final model, we identified multiple biosecurity measures which were associated with reduced HPAI spillover risk. Our objectives were to 1) identify environmental correlates associated with HPAI H5N1 spillover from wild bird populations into domestic poultry operations, 2) account for spatial and temporal trends in spillover events related to migratory bird species distributions, and 3) assess how biosecurity measures are associated with decreased instances of spillover after controlling for local factors. Models such as these can be crucial tools for predicting HPAI risk to agricultural, human, and wild systems and can be broadly applied to inform the planning of biosecurity, surveillance, and response.

## 2 Methods

### 2.1 Spatiotemporal spillover dataset

We used information from a subset of farms from the on-going HPAI H5N1 outbreak in the U.S. for which phylogenetic and epidemiologic analyses provided evidence of likely independent introductions of HPAI from wild birds into poultry systems [13]. Of HPAI spillover events which occurred during the period of interest (February 7, 2022, through January 6, 2023), 550 were identified as originating from wild bird populations (as opposed to farm-to-farm spread) and retained as case locations within our model (Fig. S1). Of these operations, 283 were classified as backyard chicken, 75 as backyard turkey, 37 as commercial chicken, and 155 as commercial turkey. In addition to location and estimated date of first infection, we also retained information on the species (i.e., turkey or chicken) and production type (i.e., backyard or commercial) at each operation. We did not include spillover events for operations not classified as turkey or chicken farms nor from case farms that had viral introduction likely due to lateral transmission/common source introduction as determined from phylogenetic analyses [13]. For our model, we assumed there were no differences in probability of detection of spillover events across operation types, as farms were obligated to report and the likelihood of high mortality in both chickens and turkeys would result in clearly identifiable events for either class. Still, backyard operations may be underrepresented in reporting datasets [29], which would result in lower risk estimates for this group. As the true locations of poultry operations in the U.S. were not publicly available, we selected background locations for comparing spillover events according to models developed by Patyk et al. [30], which simulated likely poultry farm locations and populations using remotely sensed data products and probabilistic modeling techniques. To limit background locations to the same range as our observed spillover data, we randomly selected 10,000 locations from within a minimum convex polygon constructed from observed spillover locations and buffered it by 50 km. Patyk et al. [30] assigned farms to discrete classes of “broiler”, “pullet”, “layer”, and “turkey” to describe species of production. However, the spillover surveillance dataset described a broader range of production classes, and there were many instances where farms were classified as having mixed chicken and turkey flocks. As turkey farms may be at higher risk of spillover compared to chicken operations [28], and because there were few backyard operations with only turkeys from which to make inference, we chose to reclassify mixed turkey and chicken classes as “turkey” farms for our analysis. Background locations were classified as either turkey or chicken (i.e., poultry type) and as either commercial (≥1000 birds) or backyard (<1000 birds; i.e., production type). Of the 10,550 background points (predicted farm locations) randomly selected from Patyk et al. [30], 8726 were backyard chicken, 615 were backyard turkey, 908 were commercial chicken, and 301 were commercial turkey.

### 2.2 Landscape characteristics

To describe variation in spillover according to landscape characteristics, we identified a set of candidate covariates from previously published research on HPAI outbreaks and prevalence (Table S1) as well as waterfowl movement and resource selection (Table S2). As outbreaks occurred across the contiguous United States, we identified candidate covariates from national publicly available datasets. For covariates describing landcover type as a proportion of all types found on the landscape, we generated models at a resolution of 1 km and 10 km, representing fine and broad scale movements of waterfowl as identified from reviewing prior research (Table S2). For temporally variable covariates, we assessed variation in risk at a two-week time scale, beginning two weeks prior to the date of the first observed spillover event. For datasets available at coarser scales (>2 weeks), we interpolated data between available dates. We summarized covariates as the mean of available information from the prior two-weeks to account for lag time between occurrence and identification of a spillover event. We z-standardized all continuous covariates to facilitate comparison of coefficient values.

A full summary of covariates and data sources are available in supplemental table S3. We used shapefiles from the National Wetland Inventory [31] to estimate the distance from each location to nearest wetland and the proportion of the landscape classified as wetland. From the National Hydrography Dataset [32], we classified surface water features into flowing water (rivers and streams) and waterbodies (coastlines, reservoirs, estuaries, lakes and ponds). We then estimated the proportion of the landscape and the distance to the nearest feature classified as flowing water, waterbody, or either. From the National Landcover Database (NLCD; [33]), originally available at a 30m resolution, we separately estimated the proportion of the landscape defined as developed or agricultural land use. Developed land constituted all intensities of development described by NLCD while agricultural land consisted of pastureland and row crop. We estimated aquatic normalized difference vegetation index (NDVI; [34]) using data accessed via the “MODISTools” package [35] in program R [36], available at a 250m resolution. NDVI estimates were only available in 16-day increments, requiring interpolation to produce two-week averages for each temporal period. We quantified elevation at each location using the “elevatr” package [37] in program R. To estimate human population density, we downloaded block level U.S. Census data via the “tidycensus” package [38] in program R, and divided the estimated human population at each location by the area of the corresponding census block. We collected precipitation and mean temperature estimates using the “PRISM” package [39] in program R. We summarized precipitation and temperature at two temporal scales; a single value representing the mean of the prior winter (December 2021 through January 2022) and a temporally varying covariate averaging the two-weeks prior to an event. We included a covariate for local drought conditions as defined by the U.S. Drought Monitor [40,41], which characterized drought along an ordinal scale at two-week intervals. We modified this scale such that 0 represented no drought conditions present and 5 represented “exceptional” drought conditions. We describe waterfowl density as the expected relative abundance of waterfowl species according to eBird Status and Trends [42], available at a 3km resolution, downloaded and summarized using the “ebirdst” package [43] in program R. We downloaded available data from species within the genus *Alopochen, Anas*, *Anser, Aythya, Branta, Chloelphaga, Coscoroba, Cyanochen, Cygnus, Dendrocygna, Lophonetta, Neochen, and Tadorna*. Then, we grouped species into goose (*Anser, Branta, Coscoroba, and Cygnus*), duck, and all waterfowl species. As data was available as a weekly estimate, we used the natural log of abundance averaged for each two-week period and then summed values for all species within each group for a given time.

### 2.3 Case-Control Data

Full details of the case-control study, including methods and results are described in Patyk et al. [28]. Survey data was collected from commercial meat turkey farms which raised more than 30,000 meat turkeys annually and were confirmed positive for HPAI between January and October 2022. Case farms met the USDA's HPAI case definition [44] during the study time frame and were tested for HPAI in accordance with USDA HPAI response plans. For each case farm, 2 to 5 control farms from the same state were randomly selected to be contacted, where the first one or two farms were accepted to represent unaffected farms (i.e., did not meet the USDA's case definition for HPAI) during that same timeframe. Farms were contacted for valunteer study participation and a 24-page questionnaire was administered by trained enumerators or epidemiologists by phone or mail to a representative from each participating farm. The questions focused on farm characteristics, wild birds and wildlife, biosecurity, personnel, visitors, vehicles and equipment, and management practices. For a full breakdown of the survey questions used for biosecurity covariates, see Supplementary Table S3. Questionnaires were completed for 66 case farms and 59 control farms across 12 states.

In contrast to the spatiotemporal models, we did not restrict this analysis to single covariate hypotheses, but rather allowed for models which included multiple biosecurity practices with potentially associated impacts. For example, we hypothesized an interaction between the total number of birds on a farm and the number of barns birds were housed in, assuming increased densities of poultry in a barn would increase risk. We hypothesized treating water for poultry (e.g. chlorination) would help mitigate risk from surface water sources, modeled as an interaction between whether water for poultry was sourced from surface water and if it was treated. Similarly, we hypothesized wash station use would mitigate increasing risk associated with increased traffic at a farm, and modeled this as an interaction between the number of vehicles near barns and whether a wash station was used to clean vehicles. We hypothesized an interaction between permanent and temporary bird mitigations used on the premises, allowing for the potential that a combination of temporal practices would be more impactful than either would be on their own. We constructed a single model with independent group covariates for disposal method (i.e., incinerated versus composting, burial, and rendering) and whether a farm used a shared disposal site. We similarly constructed a single model combining independent covariates for whether a farm shared vehicles, equipment, or manure. Finally, we constructed a single model combining covariates for whether fresh litter was brought onto the farm in the 14 days prior to the reference date, whether it was heat treated, and whether wild birds had access to it prior to use. All other case control covariates were applied as a single covariate added to the null model.

### 2.4 Model descriptions

To construct models for HPAI spillover risk, we followed a step-wise model building process. We first identified relevant landscape characteristics by comparing single-covariate, single-likelihood models describing HPAI spillover risk, from which we generated a final model of *R*_*env*_ according to waterfowl habitat and regional spatiotemporal trends. After controlling for these potentially confounding local and regional drivers, we generated a joint-likelihood model combining our original dataset with case-control questionnaire data at a subset of locations. We used logistic regression to estimate the relative risk, *R,* of a HPAI spillover event occurring at a given poultry operation within the contiguous U.S. As spatiotemporal (*env*) and case-control (*CC*) datasets represented slightly different sampling methods (Case-Background versus Case-Control), we employed a joint likelihood approach to link related effects shared between datasets. We assumed that variation in *R* could be assessed at two scales; 1) *R*_*env*_, broader-scale (>1 km) environmental drivers associated with the dynamics of wild bird disease systems and 2) *R*_*CC*_, finer-scale (<1 km) effects associated with individual operations' biosecurity decisions.

We first describe *R* according to single-likelihood model using landscape, weather, and wild bird covariates (*env*), such that

$$R_{\mathrm{env}}∼\alpha_{\mathrm{PxP}}+\beta_{\mathrm{env}}X_{\mathrm{env}}+\varepsilon_{\mathrm{ST}},$$

where $\alpha_{\mathrm{PxP}}$ is a poultry/production-type specific regression intercept, $\beta_{\mathrm{env}}$ represents regression coefficients describing variation associated with environmental covariates($X_{\mathrm{env}})$, and $\varepsilon_{\mathrm{ST}}$ encompasses two latent effects to account for temporal and spatial autocorrelation respectively (described further below). Once env covariates were selected, we used a joint-likelihod model to describe risk according to spatiotemporal dynamics and biosecurity measures (*CC*),

$$R_{\mathrm{CC}}∼\alpha_{\mathrm{CC}}+\beta_{\mathrm{env}}X_{\mathrm{env}}+\beta_{\mathrm{CC}}X_{\mathrm{CC}}+\varepsilon_{\mathrm{ST}},$$

where $\alpha_{\mathrm{CC}}$ indicates a case-control dataset specific regression intercept, $\beta_{\mathrm{env}}X_{\mathrm{env}}$ and $\varepsilon_{\mathrm{ST}}$ are parameters shared with the *env* model, and $\beta_{\mathrm{CC}}$ are regression coefficients describing variation associated with farm specific biosecurity measures as identified in the case-control questionnaire ($X_{\mathrm{CC}}$).

Due to the broad spatial and temporal scale of observed spillover events, we incorporated two independent latent effect components to account for spatiotemporal autocorrelation. We first account for broad, spatial autocorrelation across months by incorporating a stochastic partial differential equation latent effect (SPDE; [45,46]) into all models. In brief, this method employs data reduction to summarize the continuous process of spatial autocorrelation in observations to a discrete mesh of points across the area of interest. Mesh points were spaced to best represent the scale at which autocorrelation was expected to occur. In this case, we chose to encompass the scale of monthly waterfowl movements (Table S2), while also balancing computational efficiency, resulting in a spacing of 111 km. The SPDE was spatiotemporal (i.e., multiple meshes, each associated with discrete temporal periods) and constructed as an autoregressive (AR1) effect, such that spatial autocorrelation was related to correlation in the previous period. Mesh points were duplicated for each temporal period, in this case six-weeks (combining sets of three two-week periods), then linked to the observation data in space and time. Additionally, we included a temporal-only autoregressive (AR1) latent effect describing bi-weekly variation in spillover risk, independent of the SPDE component, to account for nation-wide temporal trends.

We generated all candidate models describing relative risk using the “R-INLA” package [47], a method for approximating Bayesian inference in program R. We used a step-wise model selection process to identify a final *R*_*env*_ model used to produce a set of *R*_*CC*_ models for inference. We first compared our set of a priori single-covariate models describing $R_{\mathrm{env}}$ using Watanabe-Akaike Information Criterion [48] (hereafter "WAIC"), where the tope performing model had the lowest score (ΔWIAC = 0) with values increasing for more weakly supported models. Models were only considered supported if they obtained a lower WAIC score than a null model with no additional environmental covariates. For covariates that were quantified at multiple spatial or temporal scales, we only considered the top performing scale as an option for inclusion in our final model for *R*_*env*_. To limit overfitting and avoid spurious environmental covariates, we limited model complexity and only selected the top three covariates of the *R*_*env*_ models performing better than the null (if available) for inclusion in a null $R_{\mathrm{CC}}\;$model used to compare case-control covariates. We compared a priori models for biosecurity measured according to WAIC and compared coefficient estimates for relative strength of effect. We assessed the correlation of all covariates, and there was no observed strength of correlation, positive or negative, greater than 0.7, indicating adequate independence between covariates.

## 3 Results

### 3.1 Environmental spatiotemporal models (R_env_)

**Fig. 1:**
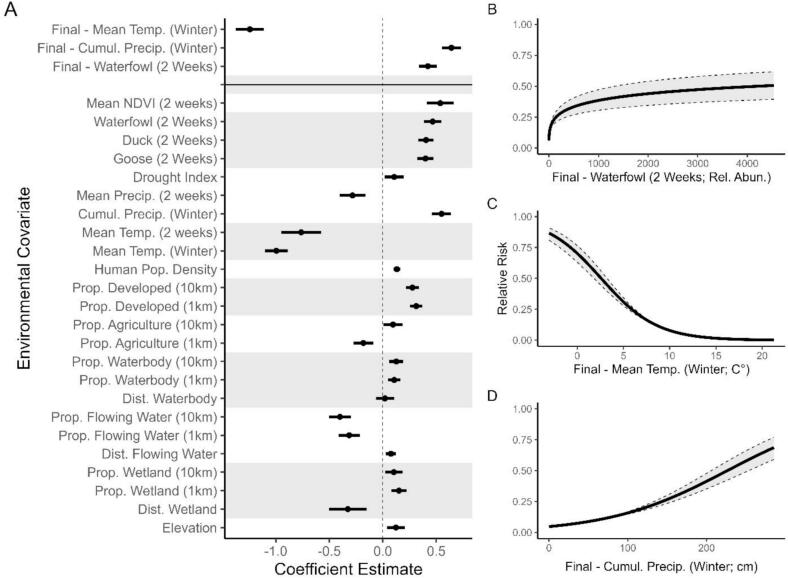
Relative strength of modeled relationships describing the relative risk of HPAI spillover according to local landscape characteristics, *R*_*env*_. All continuous covariates were *Z*-standardized to facilitate comparison of effect sizes. A) Coefficient estimates and uncertainty from a priori model describing spillover risk. Values less than 0 indicate a negative relationship, while values greater than 0 indicate a positive relationship. Grey and white stripes indicate shared model structure. B—D) Variation in relative risk across covariate space, as estimated by our final spatiotemporal model.

Our initial comparison of a priori environmental models showed all candidate models performed better than the null model (Table S4). The top performing single-covariate model describing *R*_*env*_ was based on mean temperature through the winter (Fig. 1C; ΔWAIC = 0; β = −0.994, −1.100 to −0.888 95 % C.I.). The top three performing covariates selected to produce a final $R_{\mathrm{env}}$ model included mean temperature and cumulative precipitation during winter as well as bi-weekly estimates of relative abundance of waterfowl species. The final $R_{\mathrm{env}}$ model indicated increasing risk with decreasing temperatures (β = −1.242, −1.372 to −1.112 95 % C.I.), increased precipitation (β = 0.642, 0.553–0.731 95 % C.I.), and increased waterfowl abundance (β = 0.422, 0.337–0.507 95 % C.I.).

**Fig. 2:**
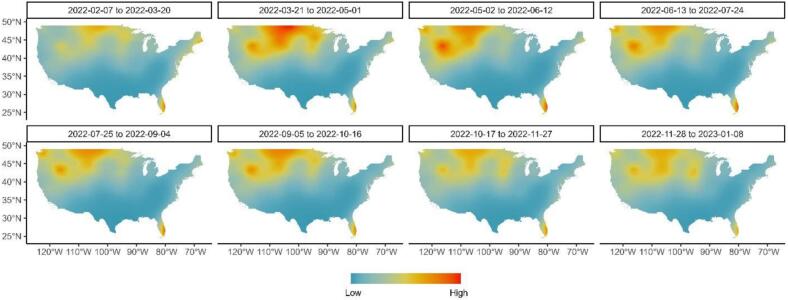
Estimates of relative risk after accounting for environmental covariates (i.e., Intercept + SPDE only) indicated regional trends in relative risk of HPAI spillover shift across the U.S. according to the time of year. Estimates were produced and presented as discrete six-week intervals.

When comparing risk among poultry production classes, we found that commercial chicken farms were 1.410 (0.998–2.024 95 % C.I.) times as likely to experience a spillover event as backyard chicken farms, compared to commercial turkey farms which were 1.497 (0.702–3.189 95 % C.I.) times as likely, and backyard turkey farms which were 2.544 (1.955–3.310 95 % C.I.) times as likely. Estimates of spatiotemporal autocorrelation in HPAI spillover risk (i.e., Intercept + SPDE only) describe variation not explaint by other covariates, and indicated regional trends shift across the U.S. according to the time of year (Fig. 2). When considering cumulative annual risk across the entire period of interest (Fig. S2B), we observed that risk was highest in the Midwest and Pacific Northwest regions of the United States, along with relatively smaller hotspots in Maine and Florida. When considering the independent temporal autoregression term, we observed two peaks in spillover risk across the United States during May and October (Fig. S2A).

### 3.2 Case-Control Questionnaire

**Fig. 3:**
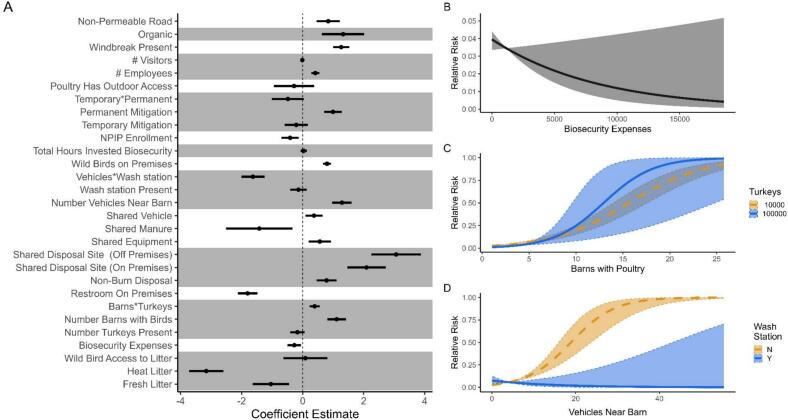
Relative strength of modeled relationships describing the relative risk of HPAI spillover according to biosecurity characteristics. All continuous covariates were Z-standardized to facilitate comparison of effect sizes. A) Coefficient estimates and uncertainty from a priori models describing spillover risk. Values less than 0 indicate a negative relationship, while values greater than 0 indicate a positive relationship. Grey and white strips indicate shared model structure. NPIP refers to the National Poultry Improvement Plan. B—D) Variation in relative risk across covariate space, as estimated by a priori case-control models.

Our comparison of a priori biosecurity models indicated support (i.e., *R*_*env*_ + Biosecurity), showing all candidate models except one performed better than the *R*_*env*_ model which was used as the null (Table S4). The single unsupported model, “Surface Water Source * Water Treatment”, did not converge and was excluded from the remaining summary of results. The top performing model describing relative spillover risk included binary covariates describing whether farms used fresh litter (i.e., bedding) (Fig. 3A; ΔWAIC = 0; β = −1.041, −1.635 to −0.447 95 % C.I.), whether fresh litter was heat treated before delivery onto the farm (β = −3.158, −3.7172 to −2.600 95 % C.I.), and whether wild birds had access to the litter before use (β = −0.089, −0.627–0.806 95 % C.I.). We again observed a dramatic decrease in WAIC scores between the top and next ranked models, which respectively supported covariates for total biosecurity expenses (Fig. 3B; ΔWAIC = −1133.73; β = −0.274, −0.494 to −0.053 95 % C.I.), an interaction between the number of barns holding birds and the number of turkeys present at a farm (Fig. 3C; ΔWAIC = −1372.72), and a model combining independent variables for whether farms shared equipment (ΔWAIC = −1581.38; β = 0.561, 0.196–0.926 95 % C.I.), vehicles (β = 0.372, 0.091–0.652 95 % C.I.), and manure or used litter (β = −1.422, −2.509 to −0.3351 95 % C.I.) with other farms. Other noteworthy models included: whether a farm used methods besides incineration for dead poultry disposal (ΔWAIC = −1618.52; β = 0.782, 0.453–1.112 95 % C.I.); if the farm shared disposal sites with other farms (on premises: β = 2.092, 1.466–2.717 95 % C.I.; off premises: β = 3.063, 2.248–3.879 95 % C.I.); an interaction between average number of vehicles near barns and whether a wash station was used (Fig. 3D; ΔWAIC = −1703.91); and whether a restroom or port-a-potty was available to crews that visited the farm (Fig. 3A; ΔWAIC = −1754.92; β = −1.806, −2.128 to −1.483 95 % C.I.).

## 4 Discussion

Maintaining best on-farm management practices around biosecurity for HPAI are crucial and even more critical with the ongoing, widespread outbreaks of HPAI in ever-expanding species, including humans. Operational and structural biosecurity for HPAI typically involves practices and standard operating procedures designed to prevent the introduction of disease into production facilities [49]. Current recommendations are based on expert opinion, epidemiological evidence, and experience in recent outbreaks. Multiple transmission pathways for HPAI virus introductions are considered, including direct interactions between wild and domestic animals, contaminated personnel and equipment entering facilities, as well as inappropriate management or disposal of manure, feed, or dead birds. Notably, after accounting for regional trends and production type, we identified multiple biosecurity practices statistically associated with reduced risk for introduction of HPAI at case farms. This may indicate that multiple transmission pathways are responsible for the different spillover events observed, and no single biosecurity effort will be wholly effective at preventing spillover.

We found that heat-treating litter (i.e., fresh bedding) was one of the most well supported biosecurity efforts for reducing risk, as HPAI virus loses infectivity when exposed to a higher temperatures for a period of time (dependent on scale; [49,50]). We also found that actions which limited contaminated employees and equipment from bringing HPAI virus into barns reduced risk. For example, in the absence of a vehicle wash station, spillover risk increased with increasing number of vehicles on premise. However, when a wash station was used, this increase in risk was dramatically mitigated. Similarly, ensuring crews had access to restrooms or port-a-potties to limit traffic around buildings reduced risk. Comparatively, wild bird mitigation efforts, such as temporary or permanent structures intended to limit access to domestic flocks or litter, did not appear to improve biosecurity. Interpretation of coefficient estimates indicate that bird mitigation was either not impactful or had slightly negative effects on risk, but this may be related to underlying data relationships and direct experimentation would be needed to fully understand how bird mitigation impacts HPAI transmission. These differing results may indicate that humans are a more significant mode of transporting HPAI into poultry operations than direct interactions between wild and domestic animals, or that wild animal controls are currently ineffective. Given the number of people and resources entering and leaving facilities daily, a broader set of biosecurity practices may be necessary to prevent contamination or spread via personnel, equipment, litter, and feed as they enter or move around facilities.

Understanding the risk of spillover at poultry farms also informs risk to wild animals, as large outbreaks in domestic flocks cause spillback into wild populations [51,52]. This cycle of spillover and spillback influences the spatiotemporal patterns of risk, as the virus is perpetuated locally, exacerbating the potential for spillover as the wild and domestic systems feedback into each other. Thus, surveillance of wild bird populations which overlap poultry farms with higher risk can be doubly effective at meeting both conservation and human health objectives. When considering poultry production broadly, we found that backyard turkey farms were the most likely production class to experience a spillover event, however this may be an artifact of treating multiple production classes within mixed backyard flocks as backyard turkey farms. We also observed a proportionally larger number of spillovers in commercial farms compared to backyard, and turkey farms experienced generally higher risk when compared to chicken farms. This may explain why areas in Minnesota experienced relatively high risk compared to what would be expected for the local landscape features, as the state had consistently high commercial turkey production [53].

Given the observed spatiotemporal relationships between waterfowl and HPAI spillover consistent with the current understanding of the HPAI system (Supplemental 1), it could be useful to combine surveillance with coarse but publicly available information on animal movements to better preempt disease transmission. For example, Birdcast (birdcast.info) is a general use tool that forecasts real time migrations of birds across the country using aerial radar systems and modeling [54]. While not currently capable of providing species-specific information, it could be used to identify when migrating birds are expected to be moving locally. Another potential key data source includes real-time telemetry data such as the conceptual AIMS for wildlife [55], which provides automated integration of real-time movement data for adaptive management. Application of such datasets in combination with HPAI surveillance and risk modeling could act as a rough early warning system. Timing reminders and training in anticipation of bird movements could spur operational awareness and limit “biosecurity fatigue” around practices which may be exhaustive to conduct at full capacity year-round. Similarly, models of bird migratory networks or connectivity among flyways [56,57] overlayed with risk maps could be used to identify the most likely transmission pathways and identify populations of most concern.

Potentially of concern, we found that multiple models related to human population (i.e., population density and development intensity) showed positive correlations with spillover risk. Although the exact cause of this relationship warrants further research, multiple studies have drawn ties between anthropogenic land use and avian influenza risk in previous outbreaks [58,59,10], with specific analyses associating road density, distance from highways, and distance from urban centers [60,61,62]. Risk associated with increased highway density may relate to the transport of poultry from farms to processing centers, which has the potential to deposit avian influenza virus along transit routes and cause spillback into wild populations [63,64]. Additionally, poultry operations in more developed areas may also inherently be forced into closer proximity with waterfowl, as they both occupy increasingly limited non-developed space on the landscape. As the barrier between wild populations and humans becomes fainter, the need for increased monitoring at these borders becomes greater.

The consistent regional increases in risk following an initial HPAI introduction may warrant additional responsive surveillance to pair with the current vigilant approach, where surveillance is immediately increased within a watershed when HPAI is identified locally. Our model provides information that may help guide additional response to existing surveillance for HPAI in the United States at the wild – poultry interface. USDA-led wild bird surveillance is organized within watershed boundaries based on expected mixing of wild bird populations and previous observations of avian influenza [65]. This year-round, national approach captures the broad extent at which spillovers were observed, across both U.S. coasts and the Midwest. Specific objectives to protect the poultry production chain may also warrant additional surveillance to be prioritized for wild bird populations that exist in agricultural areas with high spillover risk. Perhaps proportional quotas adjusted to reflect risk levels across and within watersheds would provide an efficient approach to sample such large spatial extents (i.e., contiguous United States). However, as our model was intended to identify areas of high risk at the interface between waterfowl and poultry operations, quotas set solely from these results could fail to capture broader wild bird conservation objectives, especially with the expanding species host range of HPAI [6,66]. For example, we would not expect representative sampling of seabirds, shorebirds, or habitat-generalists such as raptors and vultures [67], which have experienced large mortality events and decreased fitness when previously infected with HPAI [68,69,70]. Ideally, future efforts would work to combine the wild bird surveillance and poultry outbreak datasets into a single modeling framework to provide a more holistic representation of the disease system. Additionally, as the outbreak continues to expand to new mammalian species [71], and specifically within dairy cattle [5], it will be important to consider the broader disease system to identify other relevant data sources which could inform the spatiotemporal relationships of risk as well as continuing to identify biosecurity measures that reduce the risk of introducing avian influenza onto farms.

## CRediT authorship contribution statement

**Matthew Gonnerman:** Conceptualization, Formal analysis, Methodology, Writing – original draft. **Jennifer M. Mullinax:** Conceptualization, Methodology, Project administration, Resources, Supervision, Writing – review & editing. **Andrew Fox:** Conceptualization, Data curation, Project administration, Writing – review & editing. **Kelly A. Patyk:** Conceptualization, Data curation, Funding acquisition, Project administration, Resources, Writing – review & editing. **Victoria L. Fields:** Data curation, Project administration, Writing – review & editing. **Mary-Jane McCool:** Funding acquisition, Supervision, Writing – review & editing. **Mia K. Torchetti:** Data curation, Writing – review & editing. **Kristina Lantz:** Data curation, Writing – review & editing. **Jeffery D. Sullivan:** Conceptualization, Formal analysis, Project administration, Writing – review & editing. **Diann J. Prosser:** Conceptualization, Funding acquisition, Methodology, Project administration, Resources, Supervision, Writing – review & editing.

## Author statement

Please consider this submission for the VSI: A One Health framework for Infectious Disease Modeling. Given the One Health's stated need for submissions focused on multi-sectoral modeling, data integration, and risk assessment tools, this planned VSI would be an excellent fit for our manuscript, “Avian influenza spillover into poultry: environmental influences and biosecurity protections”. This manuscript describes a novel spatiotemporal risk model of highly pathogen avian influenza spillover from wild bird populations to domestic poultry operations. This model is constructed in a joint-likelihood framework to allow for the integration of case-control survey data describing farm biosecurity measures enacted prior to a spillover event occurring. These methods produced relevant and actionable information on both the environmental correlates of HPAI spillover events as well as the relative impact of biosecurity measures enacted during these outbreaks. Our results can broadly provide guidance on the spatiotemporal allocation of disease surveillance resource across the United States, while also informing farm operators on viable biosecurity options for reducing their risk to HPAI.

This is an original submission that has not been published and is not under consideration for publication elsewhere. All authors have read the manuscript and approve of the content and its submission. We acknowledge all of our funding sources, will receive no direct financial benefit from this publication, and have no conflicts of interest to declare. You may address any correspondence to Dr. Jennifer Mullinax and Dr. Diann Prosser. We appreciate your consideration of inclusion of our manuscript, and we look forward to your feedback.

## Declaration of competing interest

The authors declare that they have no competing interests related to this submission.

## Data Availability

The data that has been used is confidential.

## Appendix A Supplementary data

Supplementary material Image 1
