# Supplementary material for: Avian influenza spillover into poultry: environmental influences and biosecurity protections
Source: One Health. 2025 Aug 19;21:101172. doi: 10.1016/j.onehlt.2025.101172 (PMC12410409; doi:10.1016/j.onehlt.2025.101172)
Supplement: Supplementary file 1 — Supplementary material [file mmc1.docx]

SUPPLEMENTAL 1

We observed clear correlation between spillover risk and local environmental conditions, consistent with current understanding of the wild bird-HPAI disease system. Estimates of increased relative waterfowl abundance were associated with areas of higher risk, further confirming the key role that these species play in transmission (Humphreys et al., 2020; Ramey et al., 2022). As expected, winter weather was also highly correlated with spillover events, as HPAI virus can persist for longer periods of time in wet and cool conditions (Sooryanarain & Elankumaran, 2015; Vaidya & Wahl, 2015). Where these areas of increased environmental persistence overlap with increased host abundance, we expected higher spillover risk. We also observed a weak decline in risk as the proportion of area classified as streams or rivers increased within an area, consistent at multiple scales. This is opposite what would be expected given that streams and rivers frequently act as waterfowl habitat (Osborn et al., 2017; Palumbo et al., 2021), but the movement of flowing water may dilute the virus in the environment and decrease risk. Little research has focused on HPAI persistence in riverine settings (Kenmoe et al., 2024) but experimental trials in artificial streams have shown that slower moving water bodies, such as shallow streams, facilitate viral persistence in an area.

When considering the broader regional and temporal trends quantified in random effects of the models, we observed variation in risk consistent with the migratory movements of wild waterfowl populations. Temporal peaks in risk during May and October follow peaks in wild waterfowl migrations in April and October (Andersson et al., 2022; Cox et al., 2023; Stafford et al., 2014). SPDE autocorrelation estimates similarly corroborate this, although with more information to infer where birds are moving. Examining the spatiotemporal SPDE random effect estimate in isolation provides further insight into how the virus is sustained regionally (Figure 2). For example, we observed an initial but temporary risk hotspot in Maine at the beginning of the sampling period, which likely reflected the initial incursion across the Atlantic (Caliendo et al., 2022). Increased SPDE values in Florida throughout the year indicated a consistently higher level of risk than would be expected given weather conditions and estimated waterfowl abundance. As Florida is a migration hot spot (Cohen et al., 2021), this could encourage higher transmission rates as birds of all species move along the relatively narrow peninsula and deposit the virus (Poulson et al., 2020). We similarly observed a much larger, but more variable, region of increased risk in the northern Midwest and Northwest regions of the U.S. These shifting regional trends identified by the SPDE may correspond to the arrival and accumulation of infected birds, either due to migration or due to spread within wild populations. As infected birds move the virus and mix with potentially naïve populations, we expect increases in the number of infections and the extent of environmental spread locally. Similarly, migration also provides an opportunity for multiple HPAI virus subtypes to interact and reassort, resulting in new strains with potentially new disease dynamics being spread across populations (Bergervoet et al., 2019; Xie et al., 2023). It is not clear if these regional trends will be repeated in the future, as additional years of data will be necessary to monitor for such patterns. If these trends are consistent across years due to the consistent migratory patterns of waterfowl populations, then we can use these spatial patterns to further inform where and when HPAI surveillance will be most effective. However, if trends are not repeatable, our model still indicates that spillover risk increased regionally after HPAI was introduced via migrating birds.

SUPPLEMENTAL TABLES AND FIGURES


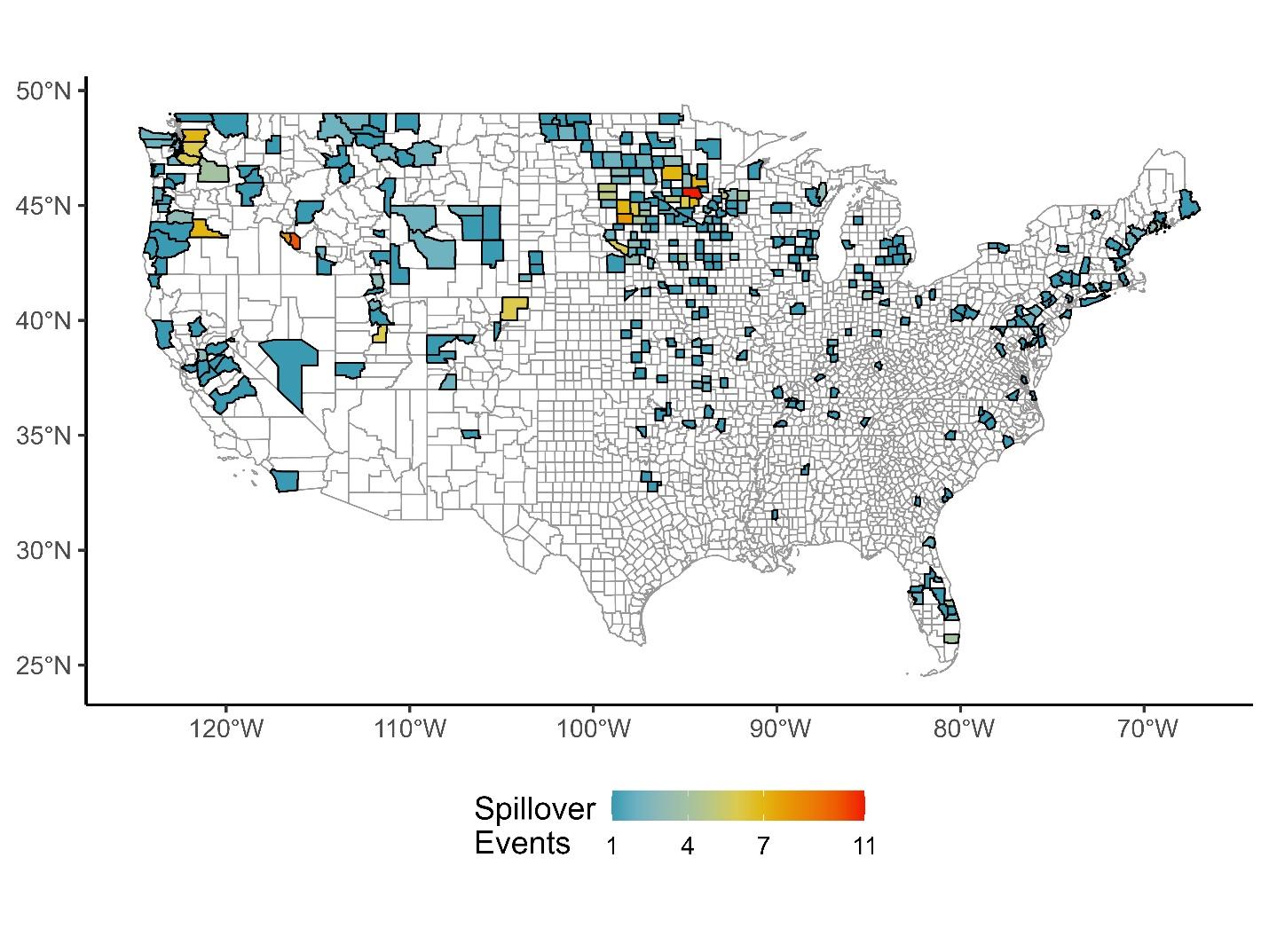
Figure S1. A map of the contiguous United States showing observed HPAI spillover from wild bird populations into domestic turkey and chicken operations as identified by phylogenetic analyses (Youk et al. 2023). We limited observations to those occurring between February 7, 2022, and January 6, 2023. Data is anonymized to be displayed at the county level, but was applied at the point level in our analysis.


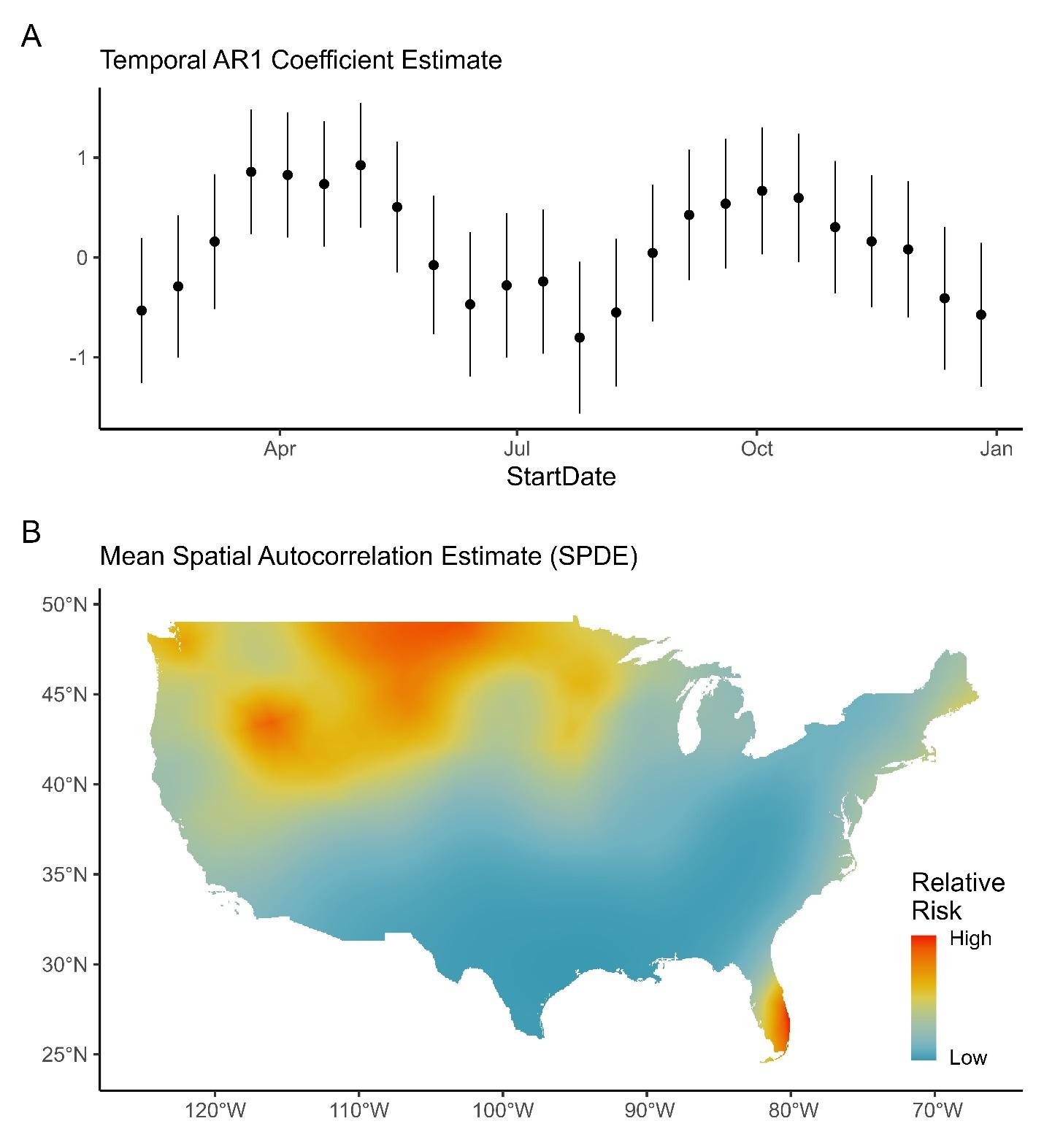


Figure S2. Spatiotemporal regression model estimates of A) temporal autocorrelation as described by an autoregressive (AR1) random effect and B) spatiotemporal autocorrelation as described by an SPDE random effect, averaged across the period February 7, 2022, through January 6, 2023. Estimates of autocorrelation indicate additional variation in risk of viral spillover from wild birds to poultry after accounting for local environmental characteristics.


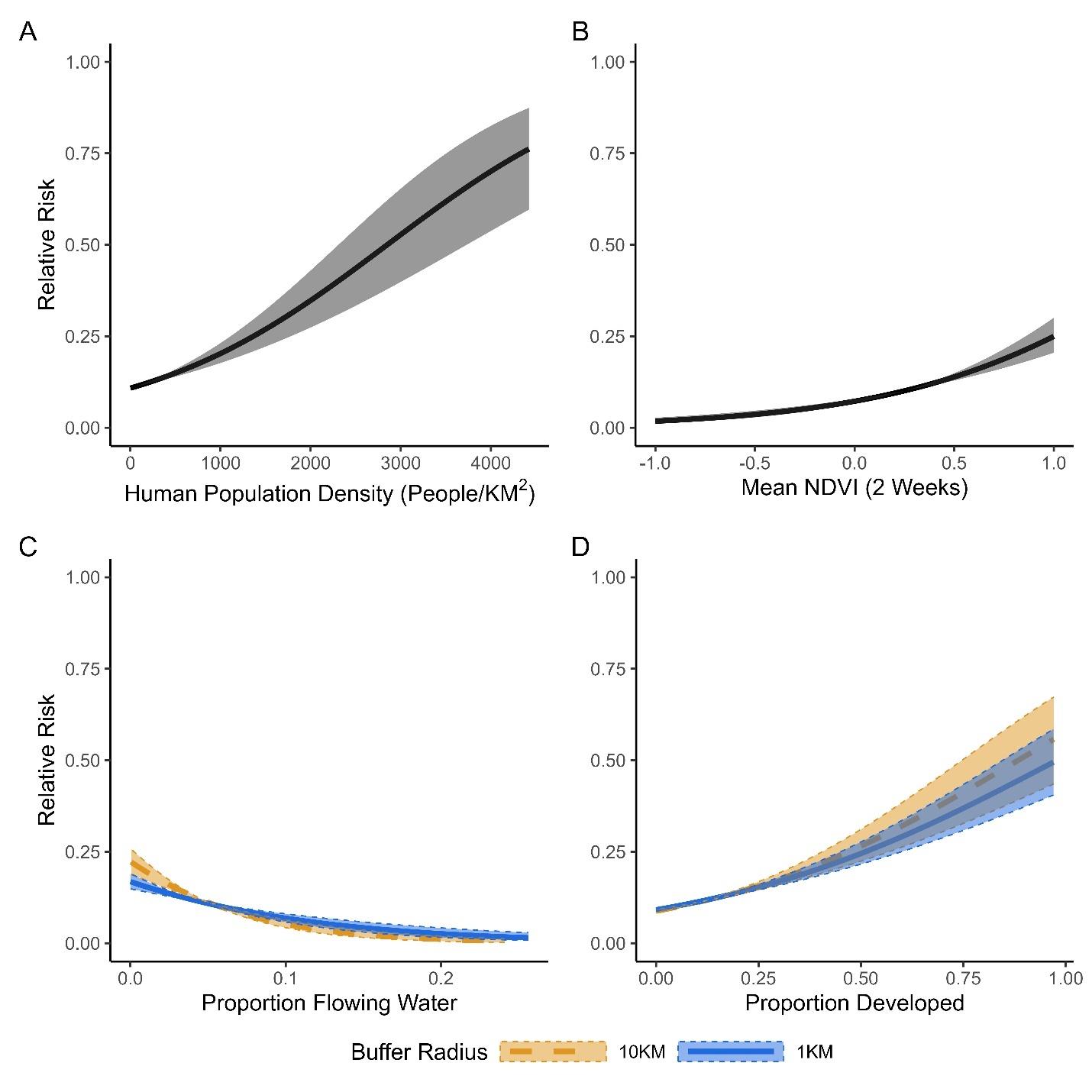


Figure S3. Relative strength of modeled relationships describing the relative risk of HPAI spillover according to local landscape characteristics. All continuous covariates were Z-standardized to facilitate comparison of effect sizes. A-D) Variation in relative risk across covariate space, as estimated by individual *a priori* environmental models.

Table S1. Table of references describing waterfowl distributions in relationship to environmental covariates.

| Type | Covariate | Publication |
| --- | --- | --- |
| Anthropogenic | Human Population Density | Martin et al. 2011 |
| Anthropogenic | Human Population Density | Adhikari et al. 2009 |
| Anthropogenic | Human Population Density | Si et al. 2013 |
| Anthropogenic | Presence of Artificial Surfaces | Scolamacchia et al. 2020 |
| Anthropogenic | Urban Area | Moriguchi et al. 2013 |
| Weather | Annual Precipitation | Herrick et al. 2013 |
| Weather | Max Temperatures in November | Si et al. 2013 |
| Weather | Mean Annual Temperature | Adhikari et al. 2009 |
| Weather | Mean Temperature in April | Herrick et al. 2013 |
| Weather | Mean Temperature in June | Herrick et al. 2013 |
| Weather | Mean Temperature in November | Herrick et al. 2013 |
| Weather | Mean Temperature of the Warmest Quarter | Alkhamis et al. 2016 |
| Weather | Mean Temperature of the Warmest Quarter | Belkhiria et al 2016 |
| Weather | Minimum Temperature | Fuller et al. 2010 |
| Weather | Minimum Temperature of Coldest Month | Belkihiria et al. 2018 |
| Weather | Precipitation | Scolamacchia et al. 2020 |
| Weather | Precipitation in November | Si et al. 2013 |
| Weather | Precipitation of Driest Quarter | Belkihiria et al. 2018 |
| Weather | Precipitation of Driest Quarter | Herrick et al. 2013 |
| Weather | Precipitation of the Warmest Quarter | Alkhamis et al. 2016 |
| Weather | Precipitation Seasonality | Belkihiria et al. 2018 |
| Weather | Thaw Date | Fuller et al. 2010 |
| Ecological | Dabbling Duck Population | Moriguchi et al. 2013 |
| Ecological | Density of Domestic Waterfowl | Martin et al. 2011 |
| Ecological | Diving Duck Population | Moriguchi et al. 2013 |
| Ecological | Waterfowl Densities/Abundance | Belkhiria et al 2016 |
| Ecological | Waterfowl Densities/Abundance | Schreuder et al. 2022 |
| Industry | Backyard Chicken Farm Density | Belkhiria et al 2016 |
| Industry | Backyard Chicken Farm Density | Belkihiria et al. 2018 |
| Industry | Broiler Farm Density | Belkihiria et al. 2018 |
| Industry | Domestic Bird Density | Pfeiffer et al. 2007 |
| Industry | Global Livestock Production System | Alkhamis et al. 2016 |
| Industry | Global Poultry Density | Alkhamis et al. 2016 |
| Industry | Poultry Density | Martin et al. 2011 |
| Industry | Poultry Density | Si et al. 2013 |
| Industry | Poultry Population Density | Scolamacchia et al. 2020 |
| Landscape | Percent Land Occupied by Water | Martin et al. 2011 |
| Landscape | Agricultural Fields | Pfeiffer et al. 2007 |
| Landscape | Agricultural Land Use | Schreuder et al. 2022 |
| Landscape | Altitude | Moriguchi et al. 2013 |
| Landscape | Areas with Tendency to Pool Water | Adhikari et al. 2009 |
| Landscape | Audubon Important Bird Areas | Belkihiria et al. 2018 |
| Landscape | Distance to Nearest Wetlands | Scolamacchia et al. 2020 |
| Landscape | Distance to Water Surfaces | Belkhiria et al 2016 |
| Landscape | Elevation | Martin et al. 2011 |
| Landscape | Elevation | Scolamacchia et al. 2020 |
| Landscape | Greenness Index | Adhikari et al. 2009 |
| Landscape | Harvested Cropland per County | Fuller et al. 2010 |
| Landscape | Latitude | Belkhiria et al 2016 |
| Landscape | NDVI | Scolamacchia et al. 2020 |
| Landscape | NDVI | Si et al. 2013 |
| Landscape | Nearest Body of Water | McDuie et al. 2022 |
| Landscape | Proximity to Lakes or Wetlands | Si et al. 2013 |
| Landscape | Slope | Adhikari et al. 2009 |

Table S2. Table of references describing waterfowl home range size across multiple scales, seasons, and methods for observing animal movements. The objectives of this review were to generate reasonable parameter values within our model, and thus this was an exploratory search not comprehensive to all published literature.

| Common | Scientific | Scale | Season | Methods | Home Range Area | Home Range Radius | Citation |
| --- | --- | --- | --- | --- | --- | --- | --- |
| Mallard | *Anas platyrhynchos* | Day | Multiple | GPS | 0.10 | 0.18 | Kleyheeg et al. 2017 |
| Mallard | *Anas platyrhynchos* | Day | Winter | GPS | 0.20 | 0.25 | van Dijk et al. 2015 |
| American Black Duck | *Anas rubripes* | Season | Breeding | VHF | 1.75 | 0.75 | Ringleman et al. 1982 |
| Green-winged Teal | *Anas crecca* | Day-to-Day | Winter | VHF | 2.01 | 0.80 | Williams et al. 2020 |
| Mallard | *Anas platyrhynchos* | Season | Winter | VHF | 4.97 | 1.26 | Legagneux et al. 2009 |
| Mallard | *Anas platyrhynchos* | Day-to-Day | Winter | VHF | 9.08 | 1.70 | Williams et al. 2020 |
| Mallard | *Anas platyrhynchos* | Diurnal | Winter | GPS | 10.18 | 1.80 | van Dijk et al. 2015 |
| American Black Duck | *Anas rubripes* | Season | Winter | GPS/VHF | 10.82 | 1.86 | Ringelman et al. 2015 |
| Canada Goose | *Branta canadensis* | Season | Breeding | Mark Resight | 16.00 | 2.26 | Rutledge et al. 2015 |
| Canada Goose | *Branta canadensis* | Season | Winter | GPS | 24.50 | 2.79 | Dorak et al. 2017 |
| Mallard | *Anas platyrhynchos* | Diurnal | Winter | VHF | 28.27 | 3.00 | Link et al. 2011 |
| Canada Goose | *Branta canadensis* | Season | Fall/Winter | GPS | 35.00 | 3.34 | Luukkonen et al. 2022 |
| American Black Duck | *Anas rubripes* | Season | Winter | VHF | 40.10 | 3.57 | Davis et al. 2022 |
| Gadwall | *Mareca strepera* | Diurnal | Winter | GPS | 45.60 | 3.81 | McDuie et al. 2019 |
| Mallard | *Anas platyrhynchos* | Season | Winter | VHF | 46.40 | 3.84 | Davis et al. 2022 |
| Mallard | *Anas platyrhynchos* | Season | Fall | GPS | 68.59 | 4.67 | Bengtsson et al. 2014 |
| Mallard | *Anas platyrhynchos* | Diurnal | Winter | GPS | 94.46 | 5.48 | McDuie et al. 2019 |
| Green-winged Teal | *Anas crecca* | Diurnal | Spring | VHF | 95.03 | 5.50 | Yetter et al. 2020 |
| Multiple | Multiple | Day-to-Day | Fall | GPS | 201.01 | 8.00 | McDuie et al. 2021 |
| Northern Pintail | *Anas acuta* | Diurnal | Winter | GPS | 202.91 | 8.04 | McDuie et al. 2019 |
| Mallard | *Anas platyrhynchos* | Season | Fall | VHF | 213.06 | 8.24 | Yetter et al. 2018 |
| Greater Snow Goose | *Anser caerulescens* | Season | Brood | VHF | 15.93 | 2.25 | Hughes et al. 1994 |
| Canada Goose | *Branta canadensis* | Season | Brood | Observations | 0.21 | 0.26 | Dunton and Combs 2010 |

Table S3. Description of all covariates used to assess HPAI spillover risk from wild bird populations to poultry operations. Covariates are presented with the model set they were included in for analysis, a description of the covariate, and the source for where it was retrieved. Acronyms: National Landcover Database (NLCD), National Wetland Inventory (NWI), National Hydrography Dataset (NHD)

| Model Set | Covariate | Description | Source |
| --- | --- | --- | --- |
| Environmental | Prop. Developed (1km) | Proportion of a 1km radius buffer around a farm location classified as developed land, as identified by the NLCD | Dewitz 2022 |
| Environmental | Prop. Developed (10km) | Proportion of a 10km radius buffer around a farm location classified as developed land, as identified by the NLCD | Dewitz 2022 |
| Environmental | Prop. Agriculture (1km)cnl | Proportion of a 1km radius buffer around a farm location classified as agricultural land, as identified by the NLCD | Dewitz 2022 |
| Environmental | Prop. Agriculture (10km) | Proportion of a 10km radius buffer around a farm location classified as agricultural land, as identified by the NLCD | Dewitz 2022 |
| Environmental | Mean Temp. (Winter) | Mean temperature estimated across the 2021-2022 winter season | Edmund et al. 2020 |
| Environmental | Cumul. Precip. (Winter) | Cumulative precipitation estimated across the 2021-2022 winter season | Edmund et al. 2020 |
| Environmental | Mean Temp. (2 weeks) | Mean temperature estimated at 2-week intervals. | Edmund et al. 2020 |
| Environmental | Mean Precip. (2 weeks) | Mean precipitation estimated at 2-week intervals. | Edmund et al. 2020 |
| Environmental | Waterfowl (2 Weeks) | Relative abundance of duck and goose species, estimated at 2-week intervals | Fink et al. 2023 |
| Environmental | Duck (2 Weeks) | Relative abundance of duck species, estimated at 2-week intervals | Fink et al. 2023 |
| Environmental | Goose (2 Weeks) | Relative abundance of goose species, estimated at 2-week intervals | Fink et al. 2023 |
| Environmental | Elevation | Location elevation | Hollister et al. 2021 |
| Environmental | Mean NDVI (2 weeks) | Aquatic Normalized Difference Vegetation Index, measured at 16-day increments | Huhkens 2022 |
| Environmental | Drought Index | Characterization of drought conditions along an ordinal scale at 2-week intervals, as described by the U.S. Drought Monitor | Svoboda et al. 2002 |
| Environmental | Dist. Wetland | Distance in meters to the nearest wetland, as identified in the NWI | USFWS 2018 |
| Environmental | Prop. Wetland (1km) | Proportion of a 1km radius buffer around a farm location classified as wetlands, as identified by the NWI | USFWS 2018 |
| Environmental | Prop. Wetland (10km) | Proportion of a 10km radius buffer around a farm location classified as wetlands, as identified by the NWI | USFWS 2018 |
| Environmental | Dist. Flowing Water | Distance in meters to the nearest river or streams, as identified in the NHD | USGS 2022 |
| Environmental | Prop. Flowing Water (1km) | Proportion of a 1km radius buffer around a farm location classified as rivers or streams, as identified by the NHD | USGS 2022 |
| Environmental | Prop. Flowing Water (10km) | Proportion of a 10km radius buffer around a farm location classified as rivers or streams, as identified by the NHD | USGS 2022 |
| Environmental | Dist. Waterbody | Distance in meters to the nearest pond or lake, as identified in the NHD | USGS 2022 |
| Environmental | Prop. Waterbody (1km) | Proportion of a 1km radius buffer around a farm location classified as ponds or lakes, as identified by the NHD | USGS 2022 |
| Environmental | Prop. Waterbody (10km) | Proportion of a 10km radius buffer around a farm location classified as ponds or lakes, as identified by the NHD | USGS 2022 |
| Environmental | Human Pop. Density | Number of humans per KM2 | Walker and Herman 2023 |
| Case-Control Questionnaire | Fresh Litter | Was fresh litter brought onto the farm during the reference period? Y/N | Patyk et al. 2023 |
| Case-Control Questionnaire | Heat Litter | Is litter heat treated prior to being delivered? Y/N | Patyk et al. 2023 |
| Case-Control Questionnaire | Wild Bird Access to Litter | Prior to use, is litter accessible by wild birds? Y/N | Patyk et al. 2023 |
| Case-Control Questionnaire | Biosecurity Expenses | Total monthly biosecurity expenses, in dollars | Patyk et al. 2023 |
| Case-Control Questionnaire | Number Turkeys Present | Number of turkeys present on the farm on the last day of the reference period | Patyk et al. 2023 |
| Case-Control Questionnaire | Number Barns with Birds | Number of barns which housed birds in the last year | Patyk et al. 2023 |
| Case-Control Questionnaire | Restroom On Premises | Was a restroom (including portable) ever provided to crews on premises? Y/N | Patyk et al. 2023 |
| Case-Control Questionnaire | Non-Burn Disposal | Did the farm use any composting, burial, rendering, or landfill disposal methods during the reference period? Y/N | Patyk et al. 2023 |
| Case-Control Questionnaire | Shared Disposal Site | Did the farm share a disposal site with another farm during the reference period? No, Yes-On Premises, Yes-Off Premises | Patyk et al. 2023 |
| Case-Control Questionnaire | Shared Equipment | Did the farm share equipment with other farms during the reference period? Y/N | Patyk et al. 2023 |
| Case-Control Questionnaire | Shared Manure | Was manure or used litter from other farms brought onto this farm prior to or during the reference period? Y/N | Patyk et al. 2023 |
| Case-Control Questionnaire | Shared Vehicle | Did the farm share vehicles with other farms during the reference period? Y/N | Patyk et al. 2023 |
| Case-Control Questionnaire | Number Vehicles Near Barn | How many vehicles per week come near the barns? | Patyk et al. 2023 |
| Case-Control Questionnaire | Wash station Present | Was a wash station or spray area used on vehicles during the reference period? Y/N | Patyk et al. 2023 |
| Case-Control Questionnaire | Wild Birds on Premises | For those water bodies within 350 yards of the farm, how many wild waterfowl or shorebirds were seen on the water during the reference period? Ordinal (orders of magnitude), 1 = None, 2 = Tens, 3 = Hundreds, 4 = Thousands | Patyk et al. 2023 |
| Case-Control Questionnaire | Total Hours Invested Biosecurity | In a typical week, how much time is spent by all employees on biosecurity activities on the farm? | Patyk et al. 2023 |
| Case-Control Questionnaire | NPIP Enrollment | Is the facility enrolled in NPIP? Y/N | Patyk et al. 2023 |
| Case-Control Questionnaire | Temporary Mitigation | Since 2015, has this farm built temporary improvements or renovations to limit wild bird access to barns? Y/N | Patyk et al. 2023 |
| Case-Control Questionnaire | Permanent Mitigation | Since 2015, has this farm built permanent improvements or renovations to limit wild bird access to barns? Y/N | Patyk et al. 2023 |
| Case-Control Questionnaire | Water Treated | Are water treatments such as chlorination used in the drinking water for the poultry on this farm? Categorical: No, Intermittently, Continuously | Patyk et al. 2023 |
| Case-Control Questionnaire | Poultry Has Outdoor Access | During the reference period, did any birds on the farm have access to the outdoors? Y/N | Patyk et al. 2023 |
| Case-Control Questionnaire | Surface Water Source | Is surface water the water source for poultry? | Patyk et al. 2023 |
| Case-Control Questionnaire | Employees | What is the total number of employees working on this farm that have access to or directly work with poultry? | Patyk et al. 2023 |
| Case-Control Questionnaire | Visitors | How many times did visitors comes to the farm during the reference period? | Patyk et al. 2023 |
| Case-Control Questionnaire | Windbreak Present | Are windbreaks present on the farm (structural, evergreen, deciduous)? Y/N | Patyk et al. 2023 |
| Case-Control Questionnaire | Organic | Is the farm certified organic? Y/N | Patyk et al. 2023 |
| Case-Control Questionnaire | Road Surface Type | Was the road surface on the farm asphalt, hard top, or gravel? Y/N | Patyk et al. 2023 |

Table S4. WAIC comparison of two model sets: Environmental and Biosecurity. Models are presented with WAIC scores and are ranked according to ΔWAIC.

| **Environmental Models** | | |  | **Biosecurity Models** | | |
| --- | --- | --- | --- | --- | --- | --- |
| Model | WAIC | ΔWAIC |  | Model | WAIC | ΔWAIC |
| Mean Temp. (Winter) | 6176.855 | 0.00 |  | Fresh Litter + Heat Litter + Wild Bird Access to Litter | 6344.727 | 0 |
| Cumul. Precip. (Winter) | 6384.380 | -207.52 |  | Biosecurity Expenses | 7478.454 | -1133.73 |
| Waterfowl (2 Weeks) | 6422.335 | -245.48 |  | Number Barns with Birds * Turkeys Present | 7717.443 | -1372.72 |
| Duck (2 Weeks) | 6424.418 | -247.56 |  | Shared Vehicle + Shared Equipment + Shared Employees | 7926.106 | -1581.38 |
| Goose (2 Weeks) | 6440.366 | -263.51 |  | Non-Burn Disposal + Shared Disposal Site | 7963.243 | -1618.52 |
| Prop. Developed (1km) | 6445.964 | -269.11 |  | Number Vehicles Near Barn * Wash Station Present | 8048.638 | -1703.91 |
| Mean NDVI (2 weeks) | 6451.221 | -274.37 |  | Wild Birds on Premises | 8067.632 | -1722.91 |
| Mean Temp. (2 weeks) | 6458.033 | -281.18 |  | Total Hours Invested Biosecurity | 8070.978 | -1726.25 |
| Prop. Developed (10km) | 6464.967 | -288.11 |  | NPIP Enrollment | 8076.68 | -1731.95 |
| Prop. Flowing Water (10km) | 6469.352 | -292.50 |  | Permanent*Temporary Mitigation | 8080.203 | -1735.48 |
| Human Pop. Density | 6487.959 | -311.10 |  | Restroom On Premises | 8099.65 | -1754.92 |
| Prop. Flowing Water (1km) | 6488.523 | -311.67 |  | Poultry Has Outdoor Access | 8103.071 | -1758.34 |
| Mean Precip. (2 weeks) | 6505.907 | -329.05 |  | Visitors + Employees | 8110.625 | -1765.9 |
| Prop. Wetland (1km) | 6511.231 | -334.38 |  | Windbreak Present | 8119.515 | -1774.79 |
| Dist. Wetland | 6511.920 | -335.06 |  | Organic | 8140.531 | -1795.8 |
| Prop. Agriculture (1km) | 6512.618 | -335.76 |  | Road Surface Type | 8192.078 | -1847.35 |
| Prop. Waterbody (1km) | 6515.386 | -338.53 |  | Null | 8211.65 | -1866.92 |
| Prop. Waterbody (10km) | 6515.498 | -338.64 |  |  |  |  |
| Elevation | 6519.126 | -342.27 |  |  |  |  |
| Dist. Flowing Water | 6519.912 | -343.06 |  |  |  |  |
| Prop. Wetland (10km) | 6520.409 | -343.55 |  |  |  |  |
| Drought Index | 6521.861 | -345.01 |  |  |  |  |
| Prop. Agriculture (10km) | 6522.688 | -345.83 |  |  |  |  |
| Null | 6524.514 | -347.66 |  |  |  |  |
